# Supplementary material for: The Size-Dependent Photonic Characteristics of Colloidal-Quantum-Dot-Enhanced Micro-LEDs
Source: Micromachines (Basel). 2023 Feb 28;14(3):589. doi: 10.3390/mi14030589 (PMC10058900; doi:10.3390/mi14030589)
Supplement: Supplementary file 1 [file micromachines-14-00589-s001.zip › micromachines-2248518-supplementary.pdf]

micromachines

Supplementary Materials

The Size-dependent Photonic Characteristics of Colloidal-Quantum-Dot-Enhanced micro-LEDs

Kai-Ling Liang, Wei-Hung Kuo and Chien-Chung Lin and Yen-Hsiang Fang

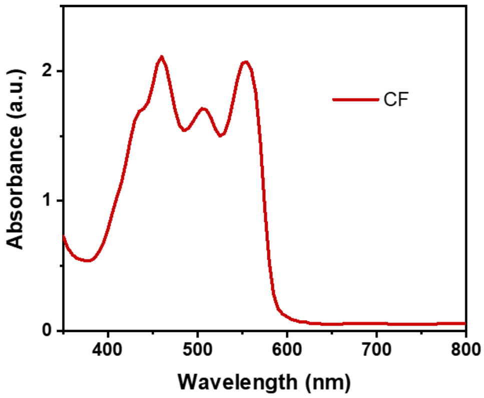

Figure S1. Absorption spectrum of color filter (CF) applied on red QD.

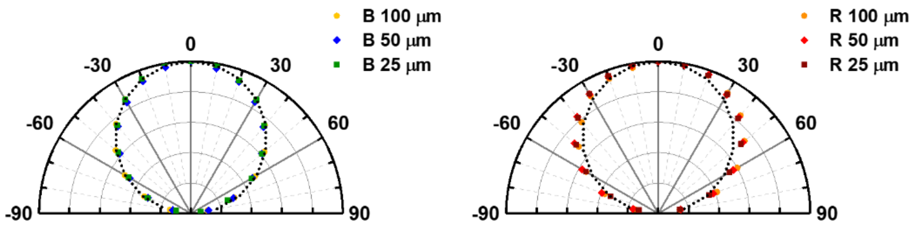

Figure S2. Measured and simulated far-field radiation of different sizes of 100 × 100, 50 × 50, and 25 × 25 μm^2 micro-LEDs in blue and red light, respectively.
